# Considerations of Fertility in Elite Sportswomen: A Narrative Review

**DOI:** 10.1111/1471-0528.18283

**Published:** 2025-07-11

**Authors:** Ariadne L'Heveder, Rachel E. Roberts, Amal Hassan, Lorraine Kasaven, Srdjan Saso, Timothy Bracewell‐Milnes, James Nicopoullos, Meen‐Yau Thum, Helen C. O'Neill, Noel Pollock, James Brown, Benjamin P. Jones

**Affiliations:** ^1^ Department of Metabolism, Digestion and Reproduction Imperial College London London UK; ^2^ Hammersmith Hospital Imperial College Healthcare NHS Trust London UK; ^3^ Institute of Sport, Exercise & Health London UK; ^4^ Lister Fertility Clinic The Lister Hospital London UK; ^5^ Genome Editing and Reproductive Genetics Group Institute for Women's Health, University College London London UK; ^6^ Research & Development Department Hertility Health Ltd. London UK; ^7^ National Performance Institute British Athletics Loughborough UK

**Keywords:** assisted reproduction, athlete, exercise, fertility, in vitro fertilisation, menstrual disorder, sportswoman, world anti‐doping agency

## Abstract

**Background:**

Increasingly more women are participating in professional and recreational sports. Whilst vigorous intensity physical activity is considered beneficial, evidence demonstrates higher rates of menstrual disturbance in elite athletes. There is less clear evidence on the impact of elite‐level exercise on fertility outcomes. It is critical to understand this given the optimal age for fertility is likely to coincide with the age of peak performance for many athletes.

**Aim:**

To perform a narrative review summarising the impact of elite‐level exercise on reproductive function and fertility outcomes, highlighting the importance of relative energy deficiency in sport (REDs). Additionally, to outline the reproductive options for female athletes and summarise the fertility treatment outcomes and challenges for athletes.

**Methods:**

A literature search of Medline, Embase, Google Scholar and Web of Science was performed.

**Results:**

There is clear evidence that elite‐level training causing increased risk of menstrual disturbance. This is likely due to REDs, with evidence that increasing energy intake can improve menstrual dysfunction. Whilst there is limited data in athletes, data from the general population suggests exercise may be beneficial for both fertility and assisted reproduction outcomes up to a certain point, beyond which there may be a negative impact, particularly if there was prior menstrual disturbance. It is crucial to be able to counsel athletes on the effect of age upon reproductive outcomes, the impact of their training on reproductive health, and the implications of fertility treatments, including the impact upon training and performance, and whether the medications involved comply with anti‐doping regulations.

**Conclusion:**

Elite‐level exercise can have a negative impact on menstrual function, however the impact on fertility is less clear. Restoring energy balance may re‐establish menstruation and enhance fertility, but some individuals may need fertility treatment. Athletes wishing to wait until after retirement to conceive may encounter age‐related reproductive decline, hence some may wish to consider oocyte/embryo preservation. Giving athletes evidence‐based information regarding their reproductive health remains challenging due to their underrepresentation in exercise‐related research.

## 1 Introduction

Women are participating in professional and recreational sports in consistently increasing numbers [1, 2]. The Paris Olympic Games 2024 saw full gender parity among athletes for the first time [2], a marked difference from 1984 when only 23% of athletes were female [3]. In their most recent guidelines, the World Health Organisation encourages partaking in vigorous intensity physical activity (VIPA) in the absence of a chronic condition [4]. VIPA is defined as any activity that requires someone to breathe hard and fast, only being able to say a few words without pausing for breath, typically elevating the heart rate to 70%–85% of the age‐predicted maximum heart rate. The exact definition of elite‐level exercise is a debated subject, with elite‐level being based on several different measures, for example, the level of competition, such as regional to international, or the amount of training. This makes comparing studies within this field challenging [5]. Regardless of the study definition, there is a great deal of evidence to demonstrate that menstrual disturbance is more common among professional athletes than non‐athletes [6]. The impact of elite‐level exercise on fertility is less clear; however, it is recognised that exercising at intense levels in non‐athlete research populations may have negative effects on fertility [7, 8, 9, 10]. This is likely related to the effect of VIPA without adequate nutrition and recovery on the hypothalamic–pituitary–ovarian (HPO) axis and therefore reproductive health [11], rather than the actual impact of the exercise alone. Notably, insufficient physical activity (PA) will also negatively impact the HPO axis and metabolic function, also potentially affecting fertility.

Importantly, the optimal age for fertility coincides with the age of peak performance for many athletes [12, 13, 14, 15, 16]. The International Olympic Committee (IOC) recognises that to date, there has been a lack of guidance in reproductive health and delaying childbearing [1]. This is particularly relevant given the increased uptake of fertility preservation and elective oocytefreezing in the public [17], on the background of a worldwide decrease in fertility rates [7, 18].

**FIGURE 1:**
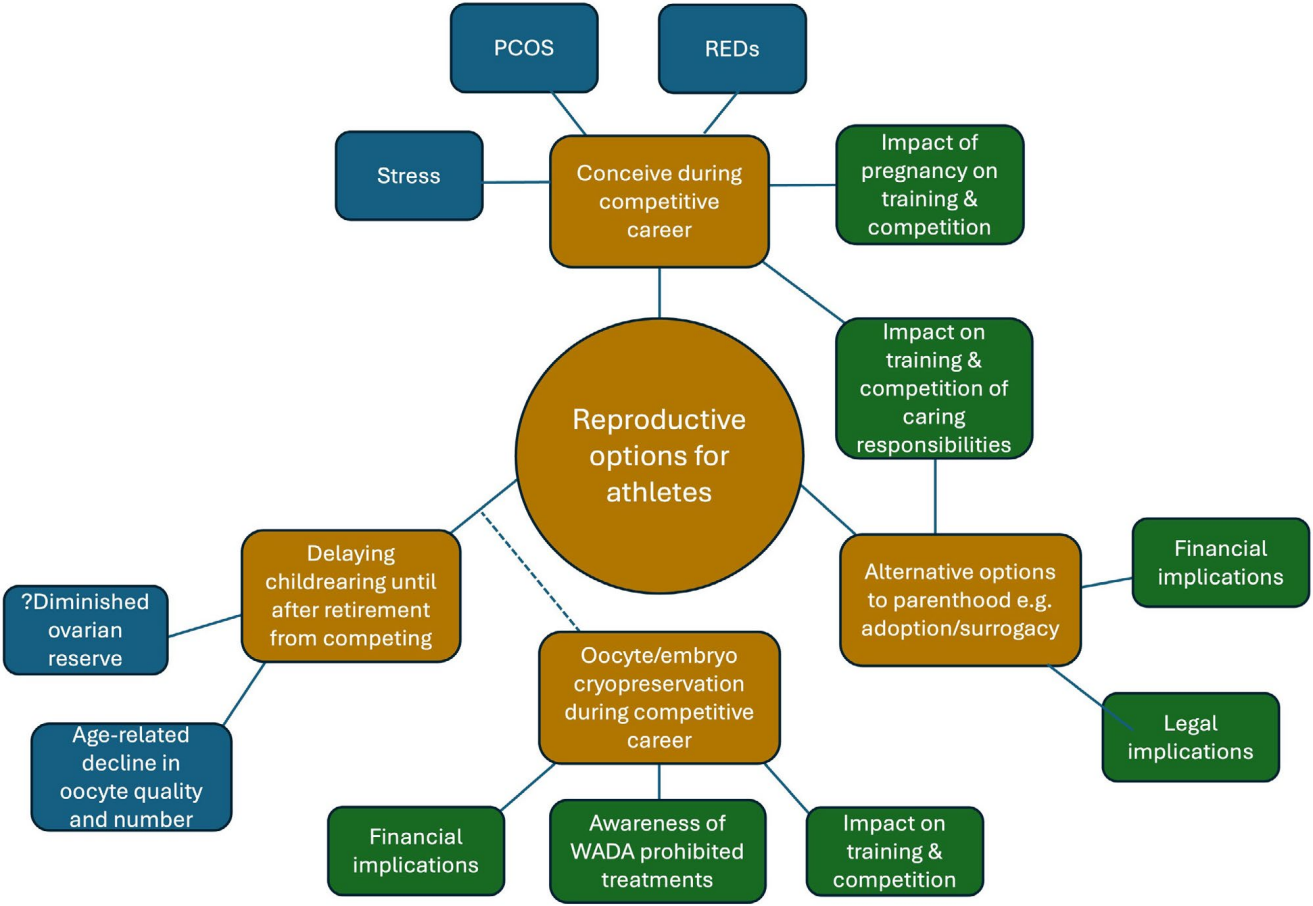
Overview of reproductive options for athletes, their implications and fertility considerations. PCOS, polycystic ovarian syndrome; REDs, relative energy deficiency in sport; WADA, world anti‐doping agency.

Despite an increasing awareness of relative energy deficiency in sport (REDs) and its impact on menstrual function, there remains a paucity of data on fertility and reproductive outcomes in elite level athletes, particularly regarding the effect of VIPA combined with lack of adequate nutrition and recovery and the impact of fertility treatment on athletic performance. It is also important to consider the potential legal and regulatory implications fertility treatment may have on competing, to support athletes, their trainers and healthcare professionals to provide optimal evidence‐based, individualised solutions for athletes. Figure 1 provides an overview of the reproductive options for athletes, their implications and the fertility considerations for each.

This narrative review aims to summarise the evidence on the impact of elite‐level exercise on reproductive function and fertility outcomes, highlighting the importance of REDs. It will address the issues of peak oocyte quality coinciding with peak athletic performance and outline the various reproductive options for female athletes. Finally, it will review fertility treatment options and challenges for athletes and summarise the evidence on assisted reproduction outcomes in female athletes. For this review, elite‐level exercise is defined as competing regionally and above, or exercising to a level comparable to that of an elite athlete.

## 2 Methods

A literature search of Medline, Embase, Google Scholar and Web of Science was performed using the following key terms: “athlete*, exercise, sport, menstruation, menstrual disturbance, fertility, assisted reproduction, in‐vitro fertilisation, oocyte OR egg freezing, relative energy deficiency in sport”. Due to the heterogeneity of definitions of elite‐level activity, where possible, the level of athlete or activity in quoted studies is described.

### 2.1 Relative Energy Deficiency in Sport

Understanding REDs is fundamental to appreciating the impact of elite‐level athleticism on reproductive function. REDs was introduced in 2014 to replace the term “Female Athlete Triad” and encompasses the interrelated health problems secondary to low energy availability (LEA) [11, 12, 19, 20]. Energy availability (EA) is calculated by subtracting energy expenditure from energy intake, relative to fat‐free mass. EA is optimal when there is adequate energy remaining to support the body to maintain health and performance [21, 22]. LEA results from a mismatch between energy intake and energy expended, and can occur with or without disordered eating [23]. Not all athletes are at equal risk of REDs; some may encounter episodes of unintended LEA, such as during periods of intense training, competitions, or in sports with high energy expenditure or involving weight restrictions [24]. Chronic LEA affects key physiological mechanisms including bone and cardiovascular health, metabolism, psychological well‐being and menstrual and reproductive function [11]. Indeed, there is an increasing body of evidence demonstrating that it is LEA, rather than high training volumes/intensities alone, that is the driver behind reproductive dysfunction [11]. REDs can negatively impact athletic performance due to reduced muscle strength, decreased endurance, loss of bone mass secondary to oestrogen deficiency, increased risk of musculoskeletal injury and psychological harm [19, 22, 25]. Despite the significant prevalence and health implications, there remains poor awareness of REDs and its associated risks among athletes and their coaches [11, 19, 22, 23]. In particular, there is still a lack of knowledge regarding safe regulation of body weight and composition [19]. Concerningly, in 2017, only 7% of international sports federations had a REDs guideline or programme, or had conducted research in this field [26]. However, there has recently been an encouraging uptake in REDs research with over 170 original research publications since the IOC published their 2018 REDs update [19]. This has resulted in an updated Physiological Model and crucially, a new REDs Clinical Assessment Tool to facilitate the detection and clinical diagnosis of REDs and provide recommendations for training and competition [19]. This will hopefully address gaps in knowledge and protect those athletes affected by, or at risk for developing REDs.

### 2.2 Menstrual Dysfunction

Numerous studies have found a higher proportion of menstrual disturbance among professional athletes compared to the general population [6, 27, 28, 29, 30, 31, 32, 33, 34]. A recent systematic review examining the prevalence of menstrual cycle disorders among female athletes not using hormonal contraception calculated a mean pooled prevalence of primary amenorrhea, secondary amenorrhea, and oligomenorrhea in athletes of 7.1% (*n* = 1260), 16% (*n* = 1705) and 23.5% (*n* = 894) respectively, with highest rates in elite/international athletes compared to highly trained/national and trained/developmental athletes [6]. These rates are higher than the prevalence of amenorrhoea and oligomenorrhoea in the general population, 3%–4% and 13.5% respectively. Menstrual disturbance is particularly common in sports where a lean body type is considered beneficial to performance [35], with 65% of long‐distance runners and 79% of ballet dancers found to be amenorrheic [30, 31]. Critically, a number of the above studies and the papers included in Taim and colleagues' systematic review date from as early as 1981. They therefore may not reflect modern training, recovery, and nutritional practices and may no longer be representative of current athletes. Nevertheless, more recent studies such as this group's work on British track and field international athletes [32], do continue to find higher levels of menstrual irregularities in professional athletes compared to the general population.

As well as oligomenorrhea/amenorrhea, other more subtle menstrual disturbances, such as anovulatory cycles and luteal phase defect (LPD) are also more common in exercising women [6, 27]. There is, however, a lack of good quality data regarding this in athletes [6]. In an observational prospective study investigating the menstrual status and daily reproductive hormone levels between sedentary (*n* = 20) and non‐athlete exercising women (*n* = 67), 95.8% of the observed menstrual cycles were ovulatory in the sedentary group compared to only 50% in the exercising group. Of the abnormal cycles in the exercising group, 29.2% were classified as LPD (short, inadequate or both) and 20.8% were classified as anovulatory [28]. Other studies have quoted the prevalence of LPD and anovulation in recreational runners to be between 38% to 64% [36, 37], and 12 to 43% respectively [12, 36]. Whilst these disturbances may be more subtle, they have profound implications for fertility. In LPD, for example, there is decreased receptivity of the endometrium for implantation of a fertilised ovum or maintenance of early pregnancy due to insufficient progesterone levels [27]. Additionally, given the heterogeneity of menstrual cycle lengths, it has been proposed that LPD may be a more sensitive marker of reproductive dysfunction [27].

Menstrual dysfunction in REDs is due to the effect of LEA on the HPO axis. LEA causes deficient pulsatile secretion of gonadotrophin‐releasing hormone (GnRH) leading to reduced luteinising hormone (LH) and follicle‐stimulating hormone (FSH) production. These lower FSH and LH levels are not sufficient to maintain normal ovulatory ovarian function, with consequent oestrogen deficiency. Table 1 summarises the hormonal changes observed secondary to REDs and LEA. There is considerable inter‐person variability in the duration and severity of LEA required to cause menstrual disturbances [22, 38], but evidence suggests that menstrual disturbance increases linearly as EA decreases [39]. Typically, the cut‐off at which EA is likely to perturb bodily functions is < 30 kcal/kg fat‐free mass (FFM)/day (125 kJ/kg FFM/day); however, this cut‐off does not predict amenorrhoea in all women [22, 39], and may not be translated to menstrual disturbance in female athletes given the threshold was established in laboratory conditions in healthy sedentary females [11].

**TABLE 1:** Reproductive implications of low energy availability. Adapted from 2023 International Olympic Committee's (IOC) consensus statement on Relative Energy Deficiency in Sport [19].

Abnormality	Evidence in sedentary women	Evidence in athletes
Alteration in LH concentrations or pulsatility	Loucks et al. 2003 [38]	Baer et al. 1993 [40]
	Loucks et al. 1998 [41]	Williams et al. 1995 [42]
	Louks et al. 2006 [43]	Rickenlund et al. 2004 [44]
	Koltun et al. 2020 [45]	Ackerman et al. 2013 [46]
	Ruffing et al. 2022 [47]	
Reduced oestrogen and progesterone	Lieberman et al. 2018 [39]	VanHeest et al. 2014 [48]
		Loucks et al. 1989 [49]
		Kaiserauer et al. 1989 [50]
		Myerson et al. 1991 [51]
		Gibbs et al. 2011 [52]
		Tornberg et al. 2017 [53]
Reduced testosterone		Rickenlund et al. 2004 [44]
Oligomenorrhea/irregular menstrual cycles	Lieberman et al. 2018 [39]	Gibbs et al. 2011 [52]
		Gibbs et al. 2013 [54]
		Hulmi et al. 2016 [55]
Primary amenorrhea		Freitas et al. 2019 [56]
		Weimann et al. 2000 [57]
Secondary amenorrhea		Kaiserauer et al. 1989 [50]
		Myerson et al. 1991 [51]
		Gibbs et al. 2011 [52]
		Gibbs et al. 2013 [54]
		Mathisen et al. 2020 [58]
		Reed et al. 2015 [59]
Luteal phase defects/Deficiency	Lieberman et al. 2018 [39]	Gibbs et al. 2013 [54]
	Koltun et al. 2020 [60]	
Anovulatory cycles	Lieberman et al. 2018 [39]	Gibbs et al. 2013 [54]

Abbreviation: LH, luteinising hormone.

Whilst functional hypothalamic amenorrhoea (FHA) is often considered the most important cause of amenorrhea among athletes, other causes of menstrual disturbance, such as polycystic ovarian syndrome (PCOS) and stress should be considered [44, 61], as well as non‐career related factors such as endometriosis. As in non‐athletes, PCOS is also a common cause for menstrual dysfunction in athletes [25], with one study of Olympic athletes finding menstrual dysfunction secondary to PCOS to be more common than FHA [29]. Whereas both cause menstrual dysfunction, the clinical picture, hormone profile and management strategies contrast significantly [62]. Notably, the two conditions can co‐present which may pose diagnostic challenges [62]. Moreover, in the context of elite sport, PCOS could enhance physical performance conferred by the hyperandrogenic hormone profile, compared to the hypo‐oestrogenic state of FHA [25, 63], including the potential for greater muscle mass and higher bone mineral density.

### 2.3 Fertility

The impact of VIPA on fertility is less well documented. Although it is recognised that moderate exercise may positively impact fertility outcomes, above a certain threshold, increasing intensity of exercise may have a negative effect particularly if there is insufficient EA [7, 8, 9, 10]. The negative impact of VIPA on female fertility is likely to be multifactorial. Exercising for ≥ 31 h per day, associated with an estimated energy expenditure of 6 kcal/min, has been associated with a 6.2‐fold increased risk of infertility [8]. The applicably of this dated study could be questioned given advances in knowledge on nutrition and rest. Additionally, exercise patterns were assessed at interview relying on participants' recall of their activity over the preceding year. A more recent 2009 prospective population‐based survey of 3887 women demonstrated that higher frequency, longer duration, and greater intensity of physical exertion were associated with subfertility [9]. In this study, women who were active on most days were 3.2 times more likely to have fertility problems than inactive women, and women exercising to exhaustion were 2.3 times more likely, compared to women doing low‐intensity activity. This clearly has implications for professional athletes who despite rest days will be active on most days. Another observational prospective cohort study of 3628 women trying to conceive, assessed the influence of PA on time to pregnancy and fecundability rates. A dose–response inverse association was observed between VIPA and fecundability, with the fecundability ratio defined as the cycle‐specific probability of conception among exposed women divided by that among unexposed women, which was maintained within subgroups for age, parity and cycle regularity, but, interestingly, not among women with a body mass index (BMI) ≥ 25 kg/m^2^ [10]. It also found that lean women who substitute VIPA with moderate PA may improve their fertility. Both studies rely on questionnaires to assess PA, which whilst validated, will inherently include measurement inaccuracies and limit the quality and reliability of the findings. A recent meta‐analysis, including seven prospective cohort studies in the general population demonstrated that high‐intensity PA, equating to a metabolic equivalent of task (MET) score or ≥ 3000 if the studies included this measure, or VIPA with high frequency or moderate PA for a long time weekly, enough to make people feel tired and weak and of a level that it has a “certain impact on normal life”, was negatively correlated with fertility [odds ratio (OR) = 0.84; 95% confidence interval (CI) 0.70–1.00, *I* ^2^ = 64%] [7]. There was, however, no such association between moderate intensity PA (> 500 MET but < 3000, or a level of PA with no impact on normal life) and fertility (OR = 1.09; 95% CI 0.98, 1.22, *I* ^2^ = 60%). Whilst the authors do not comment on the individual included studies and whether there was any assessment of EA or any reference to nutrition or recovery, they do however discuss LEA as a mechanism for their findings.

Evidence exists to suggest a positive impact of both moderate and, in contrast to the above, intense PA on fertility outcomes. In the aforementioned 2009 prospective population‐based survey, there was a decreased risk of infertility in those whose exercise was moderate (16–30 and 30–60 min compared with the shortest duration, < 15 min). Additionally, women with the highest subjective intensity of PA, rated as exercising “to exhaustion” at baseline, had the lowest frequency of continuing nulliparity and the highest frequency of having ≥ 3 children during follow‐up (*p* < 0.05) [9]. Similarly, the study of 3628 women trying to conceive found a weak positive association between moderate PA of ≥ 5 h per week and fecundability compared to low PA of < 1 h per week (≥ 5 vs. < 1 h per week: fecundability ratio 1.18, 95% CI 0.98–1.43) after adjustment in both groups for PA types [10]. A large prospective study demonstrated that an increase in vigorous activity (hiking, running, cycling, aerobic dancing, swimming or tennis ≥ 7 h per week) was associated with a reduced relative risk of ovulatory infertility [64]. This effect was time‐dependent, with each hour per week of vigorous activity, up to 7 h, associated with a 7% lower relative risk (RR) of ovulatory infertility (95% CI 4%–10%). Even when adjusted for BMI, there remained a 5% reduced risk of ovulatory infertility. Whilst the exercise was classified as vigorous, the duration per week is not extrapolatable to elite athletes, who will typically exercise well over 7 h per week [65], albeit at varying levels of intensity. Evidently other factors such as smoking status, other health conditions and alcohol consumption need to be accounted for as these may also have an impact on fertility, particularly in the general population. These may however be less pertinent in professional athletes who may not have such variability in these factors by the nature of their career choice, but are all likely to be at risk of REDs to varying degrees.

Studies focusing on fertility outcomes specifically among elite‐level athletes also show conflicting findings and are often poor quality with small cohort numbers [66, 67, 68, 69]. Indeed, Davenport's group only identified 11 studies of “low” and “very low” quality, primarily due to their observational nature, in their meta‐analysis on pregnancy outcomes in elite athletes [70]. A cross‐sectional study of 137 long‐distance runners competing at a national level found that just 8% required treatment for infertility [66]. Whilst this appears lower than the background risk of infertility in the general population where one in seven couples are infertile [71], the main factors related to infertility treatment were age at the onset of pregnancy (*p* = 0.047) and higher BMI during their athletic career (*p* = 0.032; OR 2.19). Crucially, amenorrhea or being underweight during their athletic career was not associated with reported problems related to conception. Findings from a survey of Japanese professional athletes who became pregnant after retirement (*n* = 613) found the fertility treatment rate was 11.9%, with significantly higher rates of fertility treatment in athletes with abnormal than normal menstrual cycles (17.1% vs. 10.2%, *p* = 0.0225). Multivariable logistic regression analysis demonstrated that maternal age (adjusted [OR] 1.194; 95% CI 1.129–1.262) and abnormal menstrual cycles were the relevant factors for infertility treatment [67]. A retrospective cohort study found no difference in fertility levels between 34 Norwegian athletes and 34 matched physically active controls (> 150 min per week for two years) [68]. The authors concluded that elite‐level activity does not impair fertility but only included athletes who eventually became pregnant in the five preceding years, automatically excluding athletes with long‐term sub/infertility, which may have been a consequence of their intense exercise regimes. Interestingly, the athletes highlighted a lack of advice related to energy and nutrition intake. Whilst the authors compared rates of body dissatisfaction and desire for thinness pre and post‐pregnancy, as well as rates of eating disorders prior to or during pregnancy (present in 12% athletes and 3% of controls), there was no assessment of the athletes' and controls' EA/nutrition intake. A study of 30 pregnant elite Finnish endurance athletes found 23% had preceding amenorrhea or oligomenorrhea; however, the mean time interval from ending contraception to the beginning of pregnancy was only 1.7 months (range 0–7) [69]. The authors concluded that menstrual disturbance does not affect fertility, but again, this study is subject to considerable selection bias, as only athletes who became pregnant were included. Again, there was no analysis of EA or comment on nutrition or recovery, although the authors do acknowledge that exercise‐associated infertility is usually reversible with reduction of training or weight gain, addressing that inadequate nutrition plays a key role. All studies involved self‐reported evaluation of PA either by questionnaire [66, 67, 69] or interview [68], again limiting the accuracy and reliability of the evidence. Clearly, more high‐quality data on the impact of elite‐level training on fertility is required, with a particular focus on the role of nutrition and adequate recovery, especially in athletes with menstrual disturbance.

### 2.4 Conflict Between Peak Ovarian Reserve and Oocyte Quality and Peak Athletic Performance

There is a wealth of evidence to demonstrate increasing age reduces both the chance of spontaneous pregnancy and the success of assisted reproductive techniques (ART), as well as increasing the rate of pregnancy loss, due to the reduction in ovarian reserve and oocyte quality [72, 73, 74, 75]. This is due to an exponential decrease in oocyte quality and quantity from the mid‐thirties onwards [72, 73, 74, 75]. An American prospective cohort study which recruited women between 30 and 44 years, attempting to conceive for ≤ 3 months with no known history of infertility, PCOS or endometriosis found a significant reduction in fecundity with age. Compared to women aged 30 to 31, fecundability rates were reduced by 14% in women aged 34–35 (fecundability ratio [FR] 0.86, 95% [CI] 0.68–1.08), increasingly steadily up to a 59% reduction in women aged 42–44 (FR 0.39, 95% CI 0.16–0.93) [73]. Given the general trend towards delaying childbearing, with the average maternal age of mothers in England and Wales increasing from 26.4 years in 1973 to a record high of 30.9 years in 2021 [76], it is unsurprising that deferring conception into the late thirties risks involuntary childlessness [13].

A major consideration for women wanting to have children is timing, and the impact pregnancy, maternity leave and motherhood may have on their career [77, 78]. These reproductive dilemmas are amplified for athletes, who often have a short career lifespan and fixed competition timings [77]. Furthermore, the optimal age for fertility is likely to coincide with the age of peak performance for many athletes [2, 77, 78], who have an increased tendency towards being affected by REDs‐related menstrual cycle changes or amenorrhea and impaired fertility [1, 13]. This is becoming more significant because the age at which female athletes reach their peak performance has increased in the last 20–30 years. The average age of an elite female tennis player and football player is now 26 years and 26.8 years compared to 20–22 years and 23 years in 1991 respectively [79]. The age of peak performance also varies by discipline, with the average age of rhythmic gymnasts (youngest), swimmers, tennis players and equestrians (oldest) at the Tokyo Olympics being 20, 22, 28 and 36 years old respectively [80]. In sports that require explosive movements or power, the peak age is lower, ranging from 20 to 30 years of age [14, 15]. Conversely, in endurance sports, the peak performance age is older at 25–35 years, with peak performance age increasing with increasing event distances [16].

There are broadly three options for female athletes wishing to have children as outlined in Figure 1; firstly, to have children during their sporting career, secondly to wait until after they have retired from competitive sport, or thirdly to choose alternative routes to parenthood, such as surrogacy or adoption. For those wishing to delay childbearing until after they have retired, some may consider oocyte or embryo‐freezing during their competing years as a means of mitigating age‐related fertility decline which they may encounter when attempting conception at a later age. A recent systematic review including 29 studies identified the key barriers and enablers experienced by elite athletes during preconception and pregnancy which are critical to understanding why athletes may make certain reproductive choices [78]. The most common barriers were lack of availability of evidence‐based information, lacking support from sports organisations and the attitudes, perceptions and beliefs of the athlete and society. The most common enablers were family support, athletes' perceptions and beliefs, and specific athletes' strategies to manage the demands of preconception and pregnancy such as modifying training.

An increasing number of athletes continue elite‐level sport participation during and following pregnancy [2]. For those who choose to proceed with starting a family during their sporting career, they would likely have a period of reduced or non‐competitive participation [77]. This may be more impactful for short distance and power athletes, whose duration of peak performance are shorter [14, 15]. A study on elite (competing in national or professional team) Scandinavian cross‐country skiers' views on motherhood to better understand how female athletes balance their priorities as they initiate, maintain and/or discontinue their role as a “maternal athlete” found those who had achieved competition success early in their careers were more likely to feel comfortable to have a family during their career with less concern regarding the risk of a potential reduction in performance, or even discontinuation of their competitive career, than those who might achieve success at a later age [77]. The impact and length of a break was a concern. Indeed, a study of world‐class marathon runners found maternity career breaks varied from nine to 94 months [81]. Notably, 26 runners (70.28%) establish their best performances after having children, at an average age of 32.20 ± 4.28, including the runner who took the longest break. This shows success is possible and even likely following childbirth. Besides the physical implications, other factors athletes may consider if choosing to have children during their sporting career is the societal stigma, financial implications and sponsorship deals and the lack of support from governing organisations [78]. It is incumbent on sport governing bodies, teams and sponsors to produce clear and supportive policies for women who choose to become pregnant during their sporting career. Encouragingly, an increasing number of International Federations have developed policies to support continued participation by pregnant athletes, as well as those who resume training and competition after childbirth [2].

Female athletes who chose to or feel they have no choice but to conceive after retirement may encounter age‐related fertility concerns [67, 77, 78], This may pose a particular issue for those with a later peak performance age and subsequent later retirement, such as endurance athletes or those participating in team events/sports. A survey of Olympic athletes, including 158 women found team sports athletes retired later than individual sports athletes (31.8 ± 5.1 versus 27.7 ± 6.8 years; *p* < 0.001) [82]. Tennis, for example, sees women competing into their thirties and in the case of Serena Williams, up to 40 [79]. Delaying childbearing until retirement could have significant consequences in terms of chances of conception due to the number and quality of eggs available at this age. However, the opportunity for a longer competing career potentially allows for more time to consider starting a family within training and competition. In a survey of 74 retired competitive Canadian national team rowers and rugby players, who competed between 1976 and 2019, the average age of retirement was 29 (±4) years [83]. Only three athletes gave birth during their competitive careers and six (8%) were unable to conceive. Of the 47 who delivered at least one child, 6 (8%) required fertility treatments. Interestingly, of the 17 respondents who had reached menopause, the mean age of first symptom onset or diagnosis of menopause was 48.2 ± 3.3 years, 2 years younger than the general Canadian population mean age of natural menopause of 51 years. Whilst this study sample is too small to make any significant conclusions, it highlights the potential issues athletes may encounter with delaying childbirth until after competition and suggests competing at a national team level may have an impact on reproductive aging. It is also important to consider obstetric implications associated with becoming pregnant at advanced maternal age [84, 85].

Some athletes may decide to undergo fertility preservation, by freezing oocytes or embryos, whilst working or during a break from competition and subsequently use them following retirement. Although the process is associated with physical risk and emotional burden, evidence suggests that a minority of women undergoing elective oocyte cryopreservation experience significant physical or emotional side effects [86]. In a cross sectional survey of 94 women from the general public who underwent elective oocyte cryopreservation, just 3% reported they were bothered to an ‘extreme’ amount due to the physical side effects of treatment and 14% were bothered ‘very much’, whereas 49% were bothered ‘a little’ or ‘not at all’ [86]. As such, it is likely that most athletes would be able to continue to train, to prevent deconditioning during a treatment cycle. However, athletes specifically performing explosive or acrobatic movements, should consider the increased risk of torsion in stimulated ovaries [87], and should amend their training accordingly during treatment cycles. Crucially, there are no clinical guidelines or sports federation policies to support sports medicine teams and athletes in making these decisions [2]. Encouragingly however, UK Sport's recent Pregnancy Guidance and Support for UK Sport Funded athletes does include a small section on assisted fertility and egg freezing [88]. It is important to consider that delaying childbearing using such techniques does not guarantee future success, and risks athletes potentially not meeting their reproductive aspirations from the finite number of oocytes stored, with a much lower chance of success with subsequent cycles at an older age. Indeed, a recent meta‐analysis which included 10 studies from 1999 to 2020 in which 8750 underwent planned oocyte cryopreservation, the live birth rate (LBR) per patient was 28% (95% CI of 0.24–0.33 (*I* ^2^ = 92%)), although this increased to 52% (95% CI 0.41–0.63, *I* ^2^ = 7%) for those who cryopreserved their oocytes ≤ 35 years old [17]. It is also important to note the low utilisation of cryopreserved oocytes. In the same study only 1517 women returned to use their oocytes with a return rate of 11.1% (± 4.7%). In the event of spontaneous conception or opting to not have a family, the treatment, its implications for training and the cost could be viewed as unnecessary. Nevertheless, data assessing regret among women electively cryopreserving oocytes demonstrated low rates of regret, with the most common reasons for regret being storing a suboptimal number of oocytes, and the associated financial expense [89].

### 2.5 Treatment of Infertility in Elite Sportswomen

In their latest statement, the IOC published a Clinical Assessment Tool outlining strategies for the prevention and treatment of REDs [19]. The IOC emphasises the need for a comprehensive team approach including sports medicine, nutrition, psychology, and sports science personnel, together with coach and family engagement. Clearly, the primary method to restore REDs‐related subfertility is to address the underlying cause and restore an equal energy balance; however, restoration of normal ovulatory function may take many months after a positive energy balance is restored [90]. Nevertheless, as demonstrated throughout this review, the fertility challenges for athletes are not limited to REDs and, given the conflict between the ages of peak reproductive potential and optimal performance, as well as the potential long‐term implications of years of VIPA, athletes require proactive consideration of their reproductive plans, and some may need to undergo fertility treatment. Additionally, as oocyte freezing is becoming more prevalent [17], more athletes may consider this as an option for increasing their chances of conception if they choose to start a family after retirement from competition. Hence it is important to understand and to be able to counsel athletes on three key aspects: the impact of age on reproductive outcomes, the influence of VIPA upon assisted reproduction outcomes and finally, the implications of fertility treatments, including the time requirements, the potential risks and impact upon training and performance, as well as the medications involved and whether they are compliant with anti‐doping regulations.

Before any fertility treatment is considered, it is important that attempts are made to correct any negative EA by increasing calorie intake and reducing energy expenditure, aiming for a BMI > 18.5 kg/m^2^ [13]. The ‘REFUEL’ randomised controlled trial (RCT) including 76 exercising women (≥ 2 h per week of purposeful exercise) with oligo‐ or amenorrhea demonstrated that a modest increase in daily energy intake (330 ± 65 kcal/day; 18% ± 4%) is sufficient to induce menstrual recovery [91]. Clearly this study is limited by its small sample size and its applicability to athletes who would be exercising at a higher level is debatable, yet it does demonstrate the impact of correcting LEA. Achieving a healthy BMI is also necessary to mitigate the potential increased risk of adverse obstetric and neonatal outcomes associated with low maternal weight [92], such as pregnancy loss, preterm labour [93], lower birth weight [94] and need for caesarean section [95].

The type of fertility treatment that may be required for elite athletes depends on the underlying cause of infertility. Athletes with REDs and FHA that have not responded to lifestyle changes may require ovulation induction. As typical agents used in ovulation induction, such as clomiphene and letrozole will not work in FHA due to low oestrogen levels, exogenous gonadotrophins should be used instead. However, in athletes with PCOS, ovulation induction with clomiphene or letrozole could be considered. It is crucial to be aware that clomiphene and letrozole are World Anti‐Doping Agency (WADA) prohibited due to their potential to increase testosterone production, which may confer a performance advantage [25, 63]. An athlete would therefore need a Therapeutic Use Exemption (TUE) certificate, which can be difficult to acquire [96], and may add further anxiety to an already stressful process. Exogenous gonadotrophins and ovulation triggers such as human chorionic gonadotrophin (HCG); however, are WADA compliant, so should be favoured where safe [97]. Notably, all forms of ovulation induction, and gonadotrophins in particular, have a risk of multiple pregnancy secondary to multi‐follicular growth, and ovarian hyperstimulation syndrome (OHSS). Additional oestrogen supplementation may be required if the endometrial lining remains thin during the process, and following ovulation, extra luteal phase support should be considered due to the increased risk of LPD seen in this population.

If ovulation induction with timed intercourse or intrauterine insemination is unsuccessful, or if the cause of infertility is not anovulatory, in vitro fertilisation (IVF) may be necessary. Controlled ovarian stimulation should be undertaken using gonadotrophins, as per standard protocols, without additional concern. Whilst the usage of medications including LH, GnRH agonists, and HCG are prohibited in men, this is not the case in women. However, there are numerous medications used as part of fertility treatments which are WADA prohibited in women, so it is essential clinicians are aware of these whilst making treatment plans for female athletes. Table 2 lists commonly used fertility treatments that are WADA‐prohibited.

**TABLE 2:** World anti‐doping agency prohibited fertility treatments [98].

S1 Anabolic agents – Testosterone – DHEA
S2 Peptide hormones, growth factors, related substances and mimetics – Growth hormone + analogues
S4 Hormone and metabolic modulators – Letrozole – Clomifene – Insulins and insulin‐mimetics
S9 Glucocorticoids – Prednisolone

Abbreviation: DHEA, dehydroepiandrosterone.

Letrozole has a growing number of clinical indications in reproductive medicine, due to its ability to reduce serum oestradiol levels by inhibiting aromatase activity. In ovarian stimulation, a recent systematic review demonstrated that letrozole co‐treatment significantly increased LBR in poor responders by 7%, with less gonadotrophin consumption [99]. In normal responders, significantly more oocytes were retrieved, but there was no impact upon LBR. Despite this potential, aromatase inhibitors such as letrozole are WADA prohibited at all times, owing to their ability to mask the side effects of anabolic steroids [97].

Other medications, such as dehydroepiandrosterone (DHEA), growth hormone (GH) and prednisolone have more limited evidence for their use, yet are worthy of discussion due to their potential athlete‐specific implications. DHEA is an androgen which is a precursor to testosterone and oestrogen. Both DHEA and testosterone usage are WADA prohibited at all times [97]. In the context of fertility treatment, DHEA has been shown to increase oocyte yield, fertilisation rates, embryo quality, and clinical pregnancy rates (CPR) in poor responders undergoing IVF [98, 100, 101], although other studies did not show such an improvement [102, 103, 104]. A 2015 Cochrane review summarised 17 RCTs, 15 of which were undertaken in poor responders, comparing DHEA or testosterone co‐treatment with placebo or no treatment [105]. DHEA and testosterone use were both associated with a higher LBR compared to placebo or no treatment (OR 1.88, 95% CI 1.3–2.71, 8 RCTs, *I* ^2^ −27%) and (OR 2.6, 95% CI 1.3–5.2, 4 RCTs, *I* ^2^ −0%, respectively). However, there was no statistically significant difference for both treatments when trials with high risk of bias were excluded. More recently, a meta‐analysis of 7 RCTs using testosterone co‐treatment in poor responders, compared to placebo or no treatment, found that testosterone co‐treatment significantly increased the number of oocytes retrieved and embryos created, as well as CPR and LBR [106].

GH has been shown to play a modulatory role in folliculogenesis. It regulates the effect of FSH on granulosa cells by increasing the synthesis of insulin‐like growth factor 1, thereby promoting follicular development and oocyte maturation. A 2010 Cochrane review including 10 RCTs, four of which specifically focused on poor responders, demonstrated a significant increase in CPR but no improvement in LBR [107]. Since then, further RCTs have been conducted in poor responders. The largest study, undertaken in 240 women who met the Bologna criteria for poor ovarian response, showed that those who had co‐treatment with GH required less stimulation, had a shorter duration of stimulation and had more oocytes collected, but there was no improvement in LBR [108]. A further double‐blinded RCT found that those who received GH were more likely to go ahead with oocyte retrieval but in the absence of any improvement in LBR [109]. However, a recent systematic review and meta‐analysis, which included 25 studies, 17 of which were in poor responders, demonstrated an improvement in endometrial thickness and morphology, as well as superior CPR and LBR, in those women who received GH supplementation [110]. Owing to its anabolic effect, GH is prohibited by WADA at all times.

Prednisolone also has a role in fertility treatment. It promotes trophoblast proliferation and invasion, normalises cytokine expression and uterine natural killer (NK) cell activity, and stimulates HCG secretion [111, 112]. The impact on NK cells is of particular interest given the association between elevated proportion and cytotoxicity of peripheral blood NK cells and infertility and IVF failure [113, 114], as well as a correlation between an increased number of uterine NK cells in the mid‐secretory phase and the occurrence of unexplained recurrent miscarriage (RM) [115, 116]. Whilst a recent Cochrane review of 16 RCTs showed no beneficial evidence of peri‐implantation corticosteroids for clinical outcomes among the general IVF population [117], a meta‐analysis of five RCTs demonstrated that prednisolone therapy improves outcomes in women with idiopathic RM (LBR: RR 1.58, 95% CI 1.23–2.02; miscarriage rate: RR 0.42, 95% CI 0.28–0.61) [118]. Results are less encouraging in those with recurrent implantation failure (RIF) with a recent RCT assessing whether 10 mg of prednisolone improved the chance of achieving a live birth in women with RIF compared to a placebo showing no difference (LBR 37.8% vs. 38.8% [95% CI −8.1%−6.1%]; RR 0.97 [95% CI 0.81−1.17]; *p* = 0.78) [119]. Importantly, all oral glucocorticoids, including prednisolone, are prohibited during competition [97].

As such, whilst there are numerous WADA prohibited treatments offered for a variety of indications during fertility treatment, consideration of the potential benefit is required to determine if they warrant TUE application, and whether it would be granted.

### 2.6 Assisted Reproduction Outcomes in Elite Sportswomen

Despite ARTs being particularly relevant to female athletes, there frustratingly remains a paucity of literature in this field. Although there is evidence that light to moderate exercise improves IVF outcomes [120, 121], this is likely to be up to a certain point, beyond which intense PA may have a negative effect. A systematic review and meta‐analysis of eight studies in the general population (*n* = 3683) investigating couples undergoing IVF found that CPR and LBR in physically active women were significantly higher than those in physically inactive women (OR = 1.96, 95% CI 1.40, 2.73, *I* ^2^ = 42% and OR = 1.95, 95% CI 1.06–3.59, *I* ^2^ = 82%, respectively) [122]. Notably, in three of the studies included in the meta‐analysis, physically active women exercised for > 2.5 h per week. However, for women who exercise more intensely, a negative association is seen. One study found that women having IVF who exercised > 4 h per week for between one and 9 years were 40% less likely to achieve a live birth, had a three‐fold increased risk of cycle cancellation, and had double the risk of miscarriage [60]. A more recent systematic review and meta‐analysis which included a further four studies in addition to Rao et al.'s meta‐analysis concluded that the effect of PA on LBR was uncertain (OR 1.15, 95% CI 0.92–1.43, *p* = 0.23, *I* ^2^ = 61%, 9 studies), but PA significantly improved CPR following ART (OR 1.39, 95% CI 1.08–1.79, *p* = 0.0009, *I* ^2^ = 68%, 10 studies) [123]. Whilst data from those exercising at the highest levels in these studies can be extrapolated to professional athletes, clearly the exact level and intensity of exercise will differ, including other confounders such as rest, sleep, and nutrition.

Regarding IVF success following retirement from competition, a cross‐sectional study was performed in former national level long‐distance runners to determine if future fertility was affected (*n* = 137) [66]. Whilst 94 athletes (66.4%) had a history of amenorrhea, only 11 participants (8.0%) received infertility treatment, which is lower than the national average for Japan at the time of publication. Of those who had treatment, six had been amenorrheic, and nine conceived (77.8%). Clearly, it is difficult to draw conclusions from one study with small participant numbers and little information on IVF outcomes; however, these findings do suggest that fertility treatment is successful for retired athletes.

Importantly, there are additional risks associated with pregnancies following IVF and pregnancies with advanced maternal age, and these must be considered with the risks and restrictions associated with pregnancies in elite athletes [124]. It is crucial to make athletes aware of these pregnancy risks when counselling them regarding their reproductive choices.

## 3 Conclusion

There is sufficient evidence to suggest that exercising at an elite level has a negative impact on menstrual function and can cause higher levels of anovulation and LPD. The impact on fertility is less clear cut. There may be a potentially detrimental effect, particularly in those athletes with menstrual disturbance, OR however, other evidence suggests no or a positive impact. Drawing conclusions from the existing data remains a challenge due to the small sample sizes and frequent reliance on study participants to self‐report exercise frequency and intensity in questionnaires or interviews. Whilst for some athletes, re‐establishing menstruation and fertility may be a matter of restoring energy balance, others may require fertility treatment. Some fertility treatment involves medications that are WADA prohibited; therefore, treatment customisation is necessary for elite sportswomen. Athletes who choose to start their family following retirement from professional sport may encounter age‐related reproductive decline. Elite sportswomen therefore face a reproductive dilemma between reproduction and competition planning. Sport governing bodies, teams, and sponsors must establish policies to support athletes in reproductive planning, as well as fostering supportive environments with flexible training requirements for athletes during fertility treatment and pregnancy. Additionally, athletes should be given evidence‐based information regarding their reproductive health. Elective oocyte or embryo cryopreservation may be considered by athletes who plan to achieve pregnancy after their sporting career. Currently, women remain underrepresented in exercise‐related research, and there is insufficient evidence regarding assisted reproductive outcomes in athletes. Further primary research should focus on athletes across a wide range of disciplines with objective measures of activity frequency and intensity, as well as rest and nutrition.

## Author Contributions

A.L.: Researched and wrote the article. R.E.R.: Contributed to the manuscript and revised the final draft. A.H.: Provided expertise, edited the article and revised the final draft. L.K.: Edited the article and revised the final draft. S.S.: Edited the article and revised the final draft. T.B.M.: Provided expertise and revised the final draft. J.N.: Provided expertise, edited the article and revised the final draft. M.Y.T.: Provided expertise and revised the final draft. H.C.O.: Provided expertise, edited the article, and revised the final draft. N.P.: Provided expertise, edited the article, and revised the final draft. J.B.: Provided expertise, edited the article, and revised the final draft. B.P.J.: Instigated the article, provided expertise, contributed to the manuscript, and revised the final draft.

## Ethics Statement

The authors have nothing to report.

## Conflicts of Interest

The authors declare no conflicts of interest.

## Data Availability

The data that support the findings of this study are available from the corresponding author upon reasonable request.

## References

[1] K. Bø , R. Artal , R. Barakat , et al., “Exercise and Pregnancy in Recreational and Elite Athletes: 2016 Evidence Summary From the IOC Expert Group Meeting, Lausanne. Part 1—Exercise in Women Planning Pregnancy and Those Who Are Pregnant,” British Journal of Sports Medicine 50 (2016): 571–589. 27127296 10.1136/bjsports-2016-096218

[2] M. H. Davenport , G. Bains , M. Hayman , C. Cai , N. S. Mkumbuzi , and T. L. McHugh , “Advancing Gender Equity in Sport: A Scoping Review of International Sport Federation Policies for Pregnant, Postpartum and Parenting Elite Athletes,” British Journal of Sports Medicine (2025): 1–11, 10.1136/bjsports-2024-109135. PMC12320609 40169236

[3] International Olympic Committee , “5th IOC World Conference on Women and Sport,” (2012), 1–78, https://stillmed.olympics.com/media/Document%20Library/OlympicOrg/IOC/What‐We‐Do/Promote‐Olympism/Women‐And‐Sport/Boxes%20CTA/5th‐IOC‐World‐Conference‐on‐Women‐and‐Sport‐Final‐Report‐Los‐Angeles‐2012.pdf.

[4] F. C. Bull , S. S. Al‐Ansari , S. Biddle , K. Borodulin , M. P. Buman , and G. Cardon , “World Health Organization 2020 Guidelines on Physical Activity and Sedentary Behaviour,” British Journal of Sports Medicine 54 (2020): 1451–1462. 33239350 10.1136/bjsports-2020-102955 PMC7719906

[5] C. Swann , A. Moran , and D. Piggott , “Defining Elite Athletes: Issues in the Study of Expert Performance in Sport Psychology,” Psychology of Sport and Exercise 16 (2015): 3–14.

[6] B. C. Taim , C. Ó Catháin , M. Renard , K. J. Elliott‐Sale , S. Madigan , and N. Ní Chéilleachair , “The Prevalence of Menstrual Cycle Disorders and Menstrual Cycle‐Related Symptoms in Female Athletes: A Systematic Literature Review,” Sports Medicine (Auckland, N.Z.) 53 (2023): 1963–1984. 37389782 10.1007/s40279-023-01871-8

[7] F. Zhao , X. Hong , W. Wang , J. Wu , and B. Wang , “Effects of Physical Activity and Sleep Duration on Fertility: A Systematic Review and Meta‐Analysis Based on Prospective Cohort Studies,” Frontiers in Public Health 10 (2022): 1029469. 36408057 10.3389/fpubh.2022.1029469 PMC9669984

[8] B. B. Green , J. R. Daling , N. S. Weiss , J. M. Liff , and T. Koepsell , “Exercise as a Risk Factor for Infertility With Ovulatory Dysfunction,” American Journal of Public Health 76 (1986): 1432–1436. 3777292 10.2105/ajph.76.12.1432 PMC1646979

[9] S. L. Gudmundsdottir , W. D. Flanders , and L. B. Augestad , “Physical Activity and Fertility in Women: The North‐Trondelag Health Study,” Human Reproduction 24 (2009): 3196–3204. 19801570 10.1093/humrep/dep337

[10] L. A. Wise , K. J. Rothman , E. M. Mikkelsen , H. T. Sorensen , A. H. Riis , and E. E. Hatch , “A Prospective Cohort Study of Physical Activity and Time to Pregnancy,” Fertility and Sterility 97 (2012): 113642.e1–113642.e4. 10.1016/j.fertnstert.2012.02.025 PMC3340509 22425198

[11] T. Stellingwerff , I. A. Heikura , R. Meeusen , et al., “Overtraining Syndrome (OTS) and Relative Energy Deficiency in Sport (RED‐S): Shared Pathways, Symptoms and Complexities,” Sports Medicine 51 (2021): 2251–2280. 34181189 10.1007/s40279-021-01491-0

[12] K. Bø , R. Artal , R. Barakat , et al., “Exercise and Pregnancy in Recreational and Elite Athletes: 2016/17 Evidence Summary From the IOC Expert Group Meeting, Lausanne. Part 4— Recommendations for Future Research,” British Journal of Sports Medicine 0 (2017): 1–3. 10.1136/bjsports-2017-098387 28947674

[13] B. P. Jones , A. L'Heveder , S. Saso , J. Yazbek , J. R. Smith , and M. Dooley , “Sports Gynaecology,” Obstetrics and Gynecology 21 (2019): 85–94.

[14] S. V. Allen and W. G. Hopkins , “Age of Peak Competitive Performance of Elite Athletes: A Systematic Review,” Sports Medicine 45, no. 10 (2015): 1431–1441. 26088954 10.1007/s40279-015-0354-3

[15] A. R. Elmenshawy , D. R. Machin , and H. Tanaka , “A Rise in Peak Performance Age in Female Athletes,” Age 37 (2015): 9795. 26022534 10.1007/s11357-015-9795-8 PMC4446456

[16] H. Tanaka and D. R. Seals , “Endurance Exercise Performance in Masters Athletes: Age‐Associated Changes and Underlying Physiological Mechanisms,” Journal of Physiology 586 (2008): 55–63. 17717011 10.1113/jphysiol.2007.141879 PMC2375571

[17] A. Hirsch , B. H. Raccah , R. Rotem , J. H. Hyman , I. Ben‐Ami , and A. Tsafrir , “Planned Oocyte Cryopreservation: A Systematic Review and Meta‐Regression Analysis,” Human Reproduction Update 30, no. 5 (2024): 558–568. 38654466 10.1093/humupd/dmae009

[18] GBD 2017 Population and Fertility Collaborators , “Population and Fertility by Age and Sex for 195 Countries and Territories, 1950–2017: A Systematic Analysis for the Global Burden of Disease Study 2017,” Lancet 392 (2018): 1995–2051. 30496106 10.1016/S0140-6736(18)32278-5 PMC6227915

[19] M. Mountjoy , K. E. Ackerman , D. M. Bailey , et al., “International Olympic Committee's (IOC) Consensus Statement on Relative Energy Deficiency in Sport (REDs),” British Journal of Sports Medicine 57 (2023): 1073–1097. 37752011 10.1136/bjsports-2023-106994

[20] M. Mountjoy , J. Sundgot‐Borgen , L. Burke , et al., “lThe IOC Consensus Statement: Beyond the Female Athlete Triad—Relative Energy Deficiency in Sport (RED‐S),” British Journal of Sports Medicine 48 (2014): 491–497. 24620037 10.1136/bjsports-2014-093502

[21] A. B. Loucks , “Energy Balance and Body Composition in Sports and Exercise,” Journal of Sports Sciences 22 (2004): 1–14. 14974441 10.1080/0264041031000140518

[22] M. Mountjoy , J. K. Sundgot‐Borgen , L. M. Burke , et al., “IOC Consensus Statement on Relative Energy Deficiency in Sport (RED‐S): 2018 Update,” British Journal of Sports Medicine 52, no. 11 (2018): 687–697. 29773536 10.1136/bjsports-2018-099193

[23] S. J. Verhoef , M. C. Wielink , E. A. Achterberg , M. R. Bonger , and S. M. T. A. Gossens , “Absence of Menstruation in Female Athletes: Why They Do Not Seek Help,” BMC Sports Science, Medicine and Rehabilitation 13 (2021): 146. 10.1186/s13102-021-00372-3 PMC8609260 34814941

[24] H. E. Cabre , S. R. Moore , A. E. Smith‐Ryan , and A. C. Hackney , “Relative Energy Deficiency in Sport (RED‐S): Scientific, Clinical, and Practical Implications for the Female Athlete,” Deutsche Zeitschrift fur Sportmedizin 73, no. 7 (2022): 225–234. 36479178 10.5960/dzsm.2022.546 PMC9724109

[25] A. L. Hirschberg , “Female Hyperandrogenism and Elite Sport,” Endocrine Connections 9 (2020): R81–R92. 32197237 10.1530/EC-19-0537 PMC7159262

[26] M. Mountjoy , A. Costa , R. Budgett , et al., “Health Promotion Through Sport: International Sport Federations' Priorities, Actions and Opportunities,” British Journal of Sports Medicine 52, no. 1 (2018): 54–60. 28701361 10.1136/bjsports-2017-097900

[27] S. Cannavò , L. Curtò , and F. Trimarchi , “Exercise‐Related Female Reproductive Dysfunction,” Journal of Endocrinological Investigation 24 (2001): 823–832. 11765055 10.1007/BF03343935

[28] M. J. De Souza , R. J. Toombs , J. L. Scheid , E. O'Donnell , S. L. West , and N. I. Williams , “High Prevalence of Subtle and Severe Menstrual Disturbances in Exercising Women: Confirmation Using Daily Hormone Measures,” Human Reproduction 25 (2010): 491–503. 19945961 10.1093/humrep/dep411

[29] M. Hagmar , B. Berglund , K. Brismar , and A. L. Hirschberg , “Hyperandrogenism May Explain Reproductive Dysfunction in Olympic Athletes,” Medicine and Science in Sports and Exercise 41 (2009): 1241–1248. 19461542 10.1249/MSS.0b013e318195a21a

[30] T. Dusek , “Influence of High Intensity Training on Menstrual Cycle Disorders in 56 Athletes,” Croatian Medical Journal 42 (2001): 79–82. 11172662

[31] S. F. Abraham , P. J. V. Beumont , I. S. Fraser , and D. Llewellyn‐Jones , “Body Weight, Exercise and Menstrual Status Among Ballet Dancers in Training,” British Journal of Obstetrics and Gynaecology 89 (1982): 507–510. 7093163 10.1111/j.1471-0528.1982.tb03649.x

[32] B. P. Jones , A. L'Heveder , C. Bishop , et al., “Menstrual Cycles and the Impact Upon Performance in Elite British Track and Field Athletes: A Longitudinal Study,” Frontiers in Sports and Active Living 6 (2024): 1296189. 38445211 10.3389/fspor.2024.1296189 PMC10912517

[33] A. L. Toriola and D. N. Mathur , “Menstrual Dysfunction in Nigerian Athletes,” BJOG: An International Journal of Obstetrics and Gynaecology 93 (1986): 979–985. 10.1111/j.1471-0528.1986.tb08020.x 3768291

[34] N. Pollock , C. Grogan , M. Perry , et al., “Bone‐Mineral Density and Other Features of the Female Athlete Triad in Elite Endurance Runners: A Longitudinal and Cross‐Sectional Observational Study,” International Journal of Sport Nutrition and Exercise Metabolism 20, no. 5 (2010): 418–426. 20975110 10.1123/ijsnem.20.5.418

[35] A. Nattiv , A. B. Loucks , M. M. Manore , et al., “Americans College of Sports Medicine Position Stand. The Female Athlete Triad,” Medicine and Science in Sports and Exercise 39 (2007): 1867–1882. 17909417 10.1249/mss.0b013e318149f111

[36] B. A. Bullen , G. S. Skinar , I. Z. Beitino , G. Von Mering , B. A. Turnbell , and J. W. McArthur , “Induction of Menstrual Disorders by Strenuous Exercise in Untrained Women,” New England Journal of Medicine 312 (1985): 1349–1353. 3990734 10.1056/NEJM198505233122103

[37] I. Z. Beitins , J. W. McArthur , B. A. Turnbull , G. S. Skrinar , and B. A. Bullen , “Exercise Induces Two Types of Human Luteal Dysfunction: Confirmation by Urinary Free Progesterone,” Journal of Clinical Endocrinology and Metabolism 72 (1991): 1350–1358. 1902847 10.1210/jcem-72-6-1350

[38] A. B. Loucks and J. R. Thuma , “Luteinizing Hormone Pulsatility Is Disrupted at a Threshold of Energy Availability in Regularly Menstruating Women,” Journal of Clinical Endocrinology and Metabolism 88 (2003): 297–311. 12519869 10.1210/jc.2002-020369

[39] J. L. Lieberman , M. J. DE Souza , D. A. Wagstaff , and N. I. Williams , “Menstrual Disruption With Exercise Is Not Linked to an Energy Availability Threshold,” Medicine and Science in Sports and Exercise 50 (2018): 551–561. 29023359 10.1249/MSS.0000000000001451 PMC5820163

[40] J. T. Baer , “Endocrine Parameters in Amenorrheic and Eumenorrheic Adolescent Female Runners,” International Journal of Sports Medicine 14 (1993): 191–195. 8325717 10.1055/s-2007-1021162

[41] A. B. Loucks , M. Verdun , and E. M. Heath , “Low Energy Availability, Not Stress of Exercise, Alters LH Pulsatility in Exercising Women,” Journal of Applied Physiology (1985) 84 (1998): 37–46. 10.1152/jappl.1998.84.1.37 9451615

[42] N. I. Williams , J. C. Young , J. W. McArthur , B. Bullen , G. S. Skrinar , and B. Turnbull , “Strenuous Exercise With Caloric Restriction: Effect on Luteinizing Hormone Secretion,” Medicine and Science in Sports and Exercise 27 (1995): 1390–1398. 8531610

[43] A. B. Loucks , “The Response of Luteinizing Hormone Pulsatility to 5 Days of Low Energy Availability Disappears by 14 Years of Gynecological Age,” Journal of Clinical Endocrinology and Metabolism 91 (2006): 3158–3164. 16720651 10.1210/jc.2006-0570

[44] A. Rickenlund , M. Thorén , K. Carlström , B. O. Von Schoultz , and A. L. Hirschberg , “Diurnal Profiles of Testosterone and Pituitary Hormones Suggest Different Mechanisms for Menstrual Disturbances in Endurance Athletes,” Journal of Clinical Endocrinology and Metabolism 89 (2004): 702–707. 14764784 10.1210/jc.2003-030306

[45] K. J. Koltun , M. J. De Souza , J. L. Scheid , and N. I. Williams , “Energy Availability Is Associated With Luteinizing Hormone Pulse Frequency and Induction of Luteal Phase Defects,” Journal of Clinical Endocrinology and Metabolism 105 (2020): 185–193. 31539053 10.1210/clinem/dgz030 PMC6938264

[46] K. E. Ackerman , K. T. Patel , G. Guereca , L. Pierce , D. B. Herzog , and M. Misra , “Cortisol Secretory Parameters in Young Exercisers in Relation to LH Secretion and Bone Parameters,” Clinical Endocrinology 78 (2013): 114–119. 22671919 10.1111/j.1365-2265.2012.04458.x PMC3443505

[47] K. M. Ruffing , K. J. Koltun , M. J. De Souza , and N. I. Williams , “Moderate Weight Loss Is Associated With Reductions in LH Pulse Frequency and Increases in 24‐Hour Cortisol With no Change in Perceived Stress in Young Ovulatory Women,” Physiology & Behavior 254 (2022): 113885. 35718216 10.1016/j.physbeh.2022.113885

[48] J. L. Van Heest , C. D. Rodgers , C. E. Mahoney , and M. J. De Souza , “Ovarian Suppression Impairs Sport Performance in Junior Elite Female Swimmers,” Medicine and Science in Sports and Exercise 46 (2014): 156–166. 23846160 10.1249/MSS.0b013e3182a32b72

[49] A. B. Loucks , J. F. Mortola , L. Girton , and S. S. C. Yen , “Alterations in the Hypothalamic‐Pituitary‐Ovarian and the Hypothalamic‐Pituitary Adrenal Axes in Athletic Women,” Journal of Clinical Endocrinology and Metabolism 68 (1989): 402–411. 2537332 10.1210/jcem-68-2-402

[50] S. Kaiserauer , A. C. Snyder , M. Sleeper , and J. Zierath , “Nutritional, Physiological, and Menstrual Status of Distance Runners,” Medicine and Science in Sports and Exercise 21 (1989): 120–125. 2709975

[51] M. Myerson , B. Gutin , M. P. Warren , et al., “Resting Metabolic Rate and Energy Balance in Amenorrheic and Eumenorrheic Runners,” Medicine and Science in Sports and Exercise 23 (1991): 15–22. 1997808

[52] J. C. Gibbs , N. I. Williams , J. L. Scheid , R. J. Toombs , and M. J. De Souza , “The Association of a High Drive for Thinness With Energy Deficiency and Severe Menstrual Disturbances: Confirmation in a Large Population of Exercising Women,” International Journal of Sport Nutrition and Exercise Metabolism 21 (2011): 280–290. 21813911 10.1123/ijsnem.21.4.280

[53] Å. B. Tornberg , A. Melin , F. M. Koivula , et al., “Reduced Neuromuscular Performance in Amenorrheic Elite Endurance Athletes,” Medicine and Science in Sports and Exercise 49 (2017): 2478–2485. 28723842 10.1249/MSS.0000000000001383

[54] J. C. Gibbs , N. I. Williams , R. J. Mallinson , J. L. Reed , A. D. Rickard , and M. J. De Souza , “Effect of High Dietary Restraint on Energy Availability and Menstrual Status,” Medicine and Science in Sports and Exercise 45 (2013): 1790–1797. 23954993 10.1249/MSS.0b013e3182910e11

[55] J. J. Hulmi , V. Isola , M. Suonpää , N. J. Järvinen , M. Kokkonen , and A. Wennerström , “The Effects of Intensive Weight Reduction on Body Composition and Serum Hormones in Female Fitness Competitors,” Frontiers in Physiology 7 (2016): 689. 28119632 10.3389/fphys.2016.00689 PMC5222856

[56] L. Freitas , T. Amorim , L. Humbert , et al., “Cortical and Trabecular Bone Analysis of Professional Dancers Using 3D‐DXA: A Case–Control Study,” Journal of Sports Sciences 37 (2019): 82–89. 29912627 10.1080/02640414.2018.1483178

[57] E. Weimann , C. Witzel , S. Schwidergall , and H. J. Böhles , “Peripubertal Perturbations in Elite Gymnasts Caused by Sport Specific Training Regimes and Inadequate Nutritional Intake,” International Journal of Sports Medicine 21 (2000): 210–215. 10834355 10.1055/s-2000-8875

[58] T. F. Mathisen , J. Heia , M. Raustøl , M. Sandeggen , I. Fjellestad , and J. Sundgot‐Borgen , “Physical Health and Symptoms of Relative Energy Deficiency in Female Fitness Athletes,” Scandinavian Journal of Medicine & Science in Sports 30 (2020): 135–147. 31593622 10.1111/sms.13568 PMC6916539

[59] J. L. Reed , M. J. De Souza , R. J. Mallinson , et al., “Energy Availability Discriminates Clinical Menstrual Status in Exercising Women,” Journal of the International Society of Sports Nutrition 12 (2015): 11. 25722661 10.1186/s12970-015-0072-0 PMC4342163

[60] S. N. Morris , S. A. Misssmer , D. W. Cramer , et al., “Effects of Lifetime Exercise on the Outcome of In Vitro Fertilization,” Obstetrics and Gynecology 108 (2006): 938–945. 17012457 10.1097/01.AOG.0000235704.45652.0b

[61] M. H. Davenport , A. Nesdoly , L. Ray , J. S. Thornton , R. Khurana , and T. L. F. McHugh , “Pushing for Change: A Qualitative Study of the Experiences of Elite Athletes During Pregnancy,” British Journal of Sports Medicine 56 (2022): 452–457. 35135828 10.1136/bjsports-2021-104755 PMC8995814

[62] M. Phylactou , S. A. Clarke , B. Patel , et al., “Clinical and Biochemical Discriminants Between Functional Hypothalamic Amenorrhoea (FHA) and Polycystic Ovary Syndrome (PCOS),” Clinical Endocrinology 95, no. 2 (2021): 239–252. 33354766 10.1111/cen.14402 PMC11497304

[63] S. La Vignera , R. A. Condorelli , R. Cannarella , Y. Duca , and A. E. Calogero , “Sport, Doping and Female Fertility,” Reproductive Biology and Endocrinology 16 (2018): 108. 30449281 10.1186/s12958-018-0437-8 PMC6241032

[64] J. W. Rich‐Edwards , D. Spiegelman , M. Garland , et al., “Physical Activity, Body Mass Index, and Ovulatory Disorder Infertility,” Epidemiology 13 (2002): 184–190. 11880759 10.1097/00001648-200203000-00013

[65] J. McKinney , J. Velghe , J. Fee , S. Isserow , and J. A. Drezner , “Defining Athletes and Exercisers,” American Journal of Cardiology 123, no. 3 (2019): 532–535. 30503799 10.1016/j.amjcard.2018.11.001

[66] Y. Fujita , E. Sasaki , K. Yoneda , et al., “Menstrual Status and Pregnancy in Former Elite Long‐Distance Runners With Menstrual Disorders,” Clinical Journal of Sport Medicine 33, no. 2 (2023): 172–178. 36633593 10.1097/JSM.0000000000001083

[67] S. Nose‐Ogura , O. Yoshino , H. Kamoto‐Nakamura , et al., “Age and Menstrual Cycle May Be Important in Establishing Pregnancy in Female Athletes After Retirement From Competition,” Physician and Sportsmedicine 52, no. 2 (2023): 175–180. 37019841 10.1080/00913847.2023.2199687

[68] J. Sundgot‐Borgen , C. Sundgot‐Borgen , G. Myklebust , N. Sølvberg , and M. K. Torstveit , “Elite Athletes Get Pregnant, Have Healthy Babies and Return to Sport Early Postpartum,” BMJ Open Sport & Exercise Medicine 5 (2019): e000652. 10.1136/bmjsem-2019-000652 PMC6887505 31803497

[69] J. Penttinen and R. Erkkola , “Pregnancy in Endurance Athletes,” Scandinavian Journal of Medicine & Science in Sports 7 (1997): 226–228. 9241028 10.1111/j.1600-0838.1997.tb00144.x

[70] J. Wowzdia , T. McHugh , J. Thornton , A. Sivak , M. F. Mottola , and M. H. Davenport , “Elite Athletes and Pregnancy Outcomes: A Systematic Review and Meta‐Analysis,” Medicine and Science in Sports and Exercise 53, no. 3 (2021): 534–542. 32925496 10.1249/MSS.0000000000002510

[71] Fertility Problems: Assessment and Treatment , “Clinical Guideline [CG156],” (2013).

[72] D. B. Dunson , D. D. Baird , and B. Colombo , “Increased Infertility With Age in Men and Women,” Obstetrics and Gynecology 103 (2004): 51–56. 14704244 10.1097/01.AOG.0000100153.24061.45

[73] A. Z. Steiner and A. M. Jukic , “Impact of Female Age and Nulligravidity on Fecundity in an Older Reproductive Age Cohort,” Fertility and Sterility 105 (2016): 1584–1588.e1. 26953733 10.1016/j.fertnstert.2016.02.028 PMC4893975

[74] J. D. Habbema , M. J. Eijkemans , H. Leridon , and E. R. te Velde , “Realizing a Desired Family Size: When Should Couples Start?,” Human Reproduction 30 (2015): 2215–2221. 26185187 10.1093/humrep/dev148 PMC4542717

[75] M. J. Faddy , R. G. Gosden , A. Gougeon , S. J. Richardson , and J. F. Nelson , “Accelerated Disappearance of Ovarian Follicles in Mid‐Life: Implications for Forecasting Menopause,” Human Reproduction 7 (1992): 1342–1346. 1291557 10.1093/oxfordjournals.humrep.a137570

[76] Office of National Statistics , “Birth Characteristics in England and Wales,” (2023), https://www.ons.gov.uk/peoplepopulationandcommunity/birthsdeathsandmarriages/livebirths/bulletins/birthcharacteristicsinenglandandwales/2021#age‐of‐parents.

[77] M. Bergström , S. A. Sæther , G. S. Solli , and K. McGawley , “Tick‐Tock Goes the Biological Clock: Challenges Facing Elite Scandinavian Mother‐Athletes,” Women in Sport and Physical Activity Journal 32, no. 1 (2023): 1–9.

[78] J. Titova , M. H. Davenport , A. Humphrys , and M. Hayman , “Barriers and Enablers Encountered by Elite Athletes During Preconception and Pregnancy: A Mixed‐Methods Systematic Review,” British Journal of Sports Medicine 18 (2025): 108380. 10.1136/bjsports-2024-108380 39197947

[79] R. Chomik and M. Jacinto , “Peak Performance Age in Sport. Arc Centre of Excellence in Population Ageing Research,” (2021), https://cepar.edu.au/sites/default/files/peak‐performance‐age‐sport.pdf.

[80] “The International Olympic Committee, Promotion of Women in Sport Through Time,” accessed 30/10/23, https://www.olympic.org.

[81] N. Forstmann , A. Maignié , Q. De Larochelambert , et al., “Does Maternity During Sports Career Jeopardize Future Athletic Success in Elite Marathon Runners?,” European Journal of Sport Science 23, no. 6 (2023): 896–903. 35703008 10.1080/17461391.2022.2089054

[82] C. L. de Subijana , L. Galatti , R. Moreno , and J. L. Chamorro , “Analysis of the Athletic Career and Retirement Depending on the Type of Sport: A Comparison Between Individual and Team Sports,” International Journal of Environmental Research and Public Health 17, no. 24 (2020): 9265. 33322365 10.3390/ijerph17249265 PMC7764278

[83] J. Thornton , C. Rosen , M. Davenport , et al., “Beyond the Medals: A Cross‐Sectional Study Exploring Retired Elite Female Athletes' Health,” BMJ Open Sport & Exercise Medicine 9, no. 1 (2023): e001479. 10.1136/bmjsem-2022-001479 PMC9835950 36643408

[84] P. A. Cavazos‐Rehg , M. J. Krauss , E. L. Spitznagel , et al., “Maternal Age and Risk of Labor and Delivery Complications,” Maternal and Child Health Journal 19 (2015): 1202–1211. 25366100 10.1007/s10995-014-1624-7 PMC4418963

[85] A. Londero , E. Rossetti , C. Pittini , A. Cagnacci , and L. Driul , “Maternal Age and the Risk of Adverse Pregnancy Outcomes: A Retrospective Cohort Study,” BMC Pregnancy and Childbirth 19 (2019): 261. 31337350 10.1186/s12884-019-2400-x PMC6651936

[86] B. P. Jones , A. Rajamanoharan , L. Kasaven , et al., “The Novel Use of Fertility Quality of Life (FertiQoL) Treatment Subscale to Assess Treatment Acceptability in Social Egg Freezing,” Human Fertility 25, no. 3 (2020): 447–455. 32883118 10.1080/14647273.2020.1815242

[87] M. Berkkanoglu , K. Coetzee , H. Bulut , and K. Ozgur , “Risk of Ovarian Torsion Is Reduced in GnRH Agonist Triggered Freeze‐All Cycles: A Retrospective Cohort Study,” Journal of Obstetrics and Gynaecology 39, no. 2 (2018): 212–217. 30230393 10.1080/01443615.2018.1479381

[88] UK Sport , “Pregnancy Guidance and Support for UK Sport Funded Athletes,” (2023), accessed 01/05/2025, https://www.uksport.gov.uk/resources/pregnancy‐guidance.

[89] B. P. Jones , L. Kasaven , A. L'Heveder , et al., “Perceptions, Outcomes, and Regret Following Social Egg Freezing in the UK; a Cross‐Sectional Survey,” Acta Obstetricia et Gynecologica Scandinavica 99, no. 3 (2020): 324–332. 31667820 10.1111/aogs.13763

[90] L. Falsetti , A. Gambera , L. Barbetti , and C. Specchia , “Long‐Term Follow‐Up of Functional Hypothalamic Amenorrhea and Prognostic Factors,” Journal of Clinical Endocrinology and Metabolism 87 (2002): 500–505. 11836275 10.1210/jcem.87.2.8195

[91] M. J. De Souza , R. J. Mallinson , N. C. S. Strock , et al., “Randomised Controlled Trial of the Effects of Increased Energy Intake on Menstrual Recovery in Exercising Women With Menstrual Disturbances: The ‘REFUEL’ Study,” Human Reproduction 36, no. 8 (2021): 2285–2297. 34164675 10.1093/humrep/deab149 PMC8487661

[92] ESHRE Capri Workshop Group , “Nutrition and Reproduction in Women,” Human Reproduction Update 12 (2006): 193–207. 16449360 10.1093/humupd/dmk003

[93] J. M. Moutquin , “Socio‐Economic and Psychosocial Factors in the Management and Prevention of Preterm Labour,” BJOG: An International Journal of Obstetrics and Gynaecology 110 (2003): 56–60. 12763113

[94] S. Koubaa , T. Hällström , C. Lindholm , and A. L. Hirschberg , “Pregnancy and Neonatal Outcomes in Women With Eating Disorders,” Obstetrics and Gynecology 105 (2005): 255–260. 15684148 10.1097/01.AOG.0000148265.90984.c3

[95] E. R. Hoffman , S. C. Zerwas , and C. M. Bulik , “Reproductive Issues in Anorexia Nervosa,” Expert Review of Obstetrics & Gynecology 6 (2011): 3403–3414. 10.1586/eog.11.31 PMC3192363 22003362

[96] L. Di Luigi , F. Pigozzi , P. Sgrò , L. Frati , A. Di Gianfrancesco , and M. Cappa , “The Use of Prohibited Substances for Therapeutic Reasons in Athletes Affected by Endocrine Diseases and Disorders: The Therapeutic Use Exemption (TUE) in Clinical Endocrinology,” Journal of Endocrinological Investigation 43 (2020): 563–573. 31734891 10.1007/s40618-019-01145-z

[97] World Anti‐doping Agency , World Anti‐Doping Code (International Standard Prohibited List, 2024), https://www.wada‐ama.org/sites/default/files/2023‐09/2024list_en_final_22_september_2023.pdf.

[98] D. Barad and N. Gleicher , “Effect of Dehydroepiandrosterone on Oocyte and Embryo Yields, Embryo Grade and Cell Number in IVF,” Human Reproduction 21, no. 11 (2006): 2845–2849. 16997936 10.1093/humrep/del254

[99] N. S. Bülow , M. Dreyer Holt , S. O. Skouby , et al., “Co‐Treatment With Letrozole During Ovarian Stimulation for IVF/ICSI: A Systematic Review and Meta‐Analysis,” Reproductive Biomedicine Online 44, no. 4 (2022): 717–736. 35183444 10.1016/j.rbmo.2021.12.006

[100] D. Barad , H. Brill , and N. Gleicher , “Update on the Use of Dehydroepiandrosterone Supplementation Among Women With Diminished Ovarian Function,” Journal of Assisted Reproduction and Genetics 24, no. 12 (2007): 629–634. 18071895 10.1007/s10815-007-9178-x PMC3454995

[101] A. Wiser , O. Gonen , Y. Ghetler , T. Shavit , A. Berkovitz , and A. Shulman , “Addition of Dehydroepiandrosterone (DHEA) for Poor Responder Patients Before and During IVF Treatment Improves the Pregnancy Rate: A Randomized Prospective Study,” Human Reproduction 25 (2010): 2496–2500. 20729538 10.1093/humrep/deq220

[102] C. Sciard , J. Berthiller , A. Brosse , et al., “Preliminary Results of DHEA in Poor Responders in IVF,” Open Journal of Obstetrics and Gynecology 6 (2016): 396–403.

[103] M. Kara , T. Aydin , T. Aran , N. Turktekin , and B. Ozdemir , “Does Dehydroepiandrosterone Supplementation Really Affect IVF‐ICSI Outcome in Women With Poor Ovarian Reserve?,” European Journal of Obstetrics, Gynecology, and Reproductive Biology 173 (2014): 63–65. 24331115 10.1016/j.ejogrb.2013.11.008

[104] T. W. Yeung , J. Chai , R. H. Li , V. C. Lee , P. C. Ho , and E. H. Ng , “A Randomized, Controlled, Pilot Trial on the Effect of Dehydroepiandrosterone on Ovarian Response Markers, Ovarian Response, and In Vitro Fertilization Outcomes in Poor Responders,” Fertility and Sterility 102 (2014): 108–115. 24796766 10.1016/j.fertnstert.2014.03.044

[105] H. E. Nagels , J. R. Rishworth , C. S. Siristatidis , and B. Kroon , “Androgens (Dehydroepiandrosterone or Testosterone) for Women Undergoing Assisted Reproduction,” Cochrane Database of Systematic Reviews 11 (2015): CD009749. 10.1002/14651858.CD009749.pub2 PMC10559340 26608695

[106] M. Noventa , A. Vitagliano , A. Andrisani , et al., “Testosterone Therapy for Women With Poor Ovarian Response Undergoing IVF: A Meta‐Analysis of Randomized Controlled Trials,” Journal of Assisted Reproduction and Genetics 36, no. 4 (2019): 673–683. 30610664 10.1007/s10815-018-1383-2 PMC6505000

[107] J. M. N. Duffy , G. Ahmad , L. Mohiyiddeen , L. G. Nardo , and A. Watson , “Growth Hormone for In Vitro Fertilization,” Cochrane Database of Systematic Reviews 1 (2010): CD000099. 10.1002/14651858.CD000099.pub3 PMC7058116 20091500

[108] D. M. R. Dakhly , Y. A. Bassiouny , Y. A. Bayoumi , M. A. Hassan , H. M. Gouda , and A. A. Hassan , “The Addition of Growth Hormone Adjuvant Therapy to the Long Down Regulation Protocol in Poor Responders Undergoing In Vitro Fertilization: Randomized Control Trial,” European Journal of Obstetrics, Gynecology, and Reproductive Biology 228 (2018): 161–165. 29957401 10.1016/j.ejogrb.2018.06.035

[109] R. J. Norman , H. Alvino , L. M. Hull , et al., “Human Growth Hormone for Poor Responders: A Randomized Placebo‐Controlled Trial Provides no Evidence for Improved Live Birth Rate,” Reproductive Biomedicine Online 38 (2019): 908–915. 30954433 10.1016/j.rbmo.2019.02.003

[110] Y. Shang , M. Wu , R. He , Y. Ye , and X. Sun , “Administration of Growth Hormone Improves Endometrial Function in Women Undergoing In Vitro Fertilization: A Systematic Review and Meta‐Analysis,” Human Reproduction Update 28, no. 6 (2022): 838–857. 35641113 10.1093/humupd/dmac028

[111] Practice Committee of the American Society for Reproductive Medicine , “Practice Committee of the American Society for Reproductive Medicine. The Role of Immunotherapy in In Vitro Fertilization: A Guideline,” Fertility and Sterility 110, no. 3 (2018): 387–400. 30098685 10.1016/j.fertnstert.2018.05.009

[112] A. E. Michael and A. T. Papageorghiou , “Potential Significance of Physiological and Pharmacological Glucocorticoids in Early Pregnancy,” Human Reproduction Update 14, no. 5 (2008): 497–517. 18552168 10.1093/humupd/dmn021

[113] N. Karami , M. G. Boroujerdnia , R. Nikbakht , and A. Khodadadi , “Enhancement of Peripheral Blood CD56 Dim Cell and NK Cell Cytotoxicity in Women With Recurrent Spontaneous Abortion or In Vitro Fertilization Failure,” Journal of Reproductive Immunology 95 (2012): 87–92. 22854126 10.1016/j.jri.2012.06.005

[114] M. Y. Thum , S. Bhaskaran , H. I. Abdalla , et al., “An Increase in the Absolute Count of CD56dimCD16+ CD69+ NK Cells in the Peripheral Blood Is Associated With a Poorer IVF Treatment and Pregnancy Outcome,” Human Reproduction 19 (2004): 2395–2400. 15319390 10.1093/humrep/deh378

[115] M. H. Lachapelle , P. Miron , R. Hemmings , and D. C. Roy , “Endometrial T, B, and NK Cells in Patients With Recurrent Spontaneous Abortion. Altered Profile and Pregnancy Outcome,” Journal of Immunology (Baltimore, Md.: 1950) 156 (1996): 4027–4034. 8621945

[116] K. Clifford , A. M. Flanagan , and L. Regan , “Endometrial CD56 Natural Killer Cells in Women With Recurrent Miscarriage: A Histomorphometric Study,” Human Reproduction 14 (1999): 2727–2730. 10548610 10.1093/humrep/14.11.2727

[117] C. M. Boomsma , M. S. Kamath , S. D. Keay , and N. S. Macklon , “Peri‐Implantation Glucocorticoid Administration for Assisted Reproductive Technology Cycles,” Cochrane Database of Systematic Reviews 6, no. 6 (2022): CD005996. 35771604 10.1002/14651858.CD005996.pub4 PMC9245898

[118] S. Dan , W. Wei , S. Yichao , et al., “Effect of Prednisolone Administration on Patients With Unexplained Recurrent Miscarriage and in Routine Intracytoplasmic Sperm Injection: A Meta‐Analysis,” American Journal of Reproductive Immunology 74 (2015): 89–97. 25753479 10.1111/aji.12373

[119] Y. Sun , L. Cui , Y. Lu , et al., “Prednisone vs Placebo and Live Birth in Patients With Recurrent Implantation Failure Undergoing In Vitro Fertilization: A Randomized Clinical Trial,” Journal of the American Medical Association 329, no. 17 (2023): 1460–1468. 37129654 10.1001/jama.2023.5302 PMC10155063

[120] S. Palomba , A. Falbo , B. Valli , et al., “Physical Activity Before IVF and ICSI Cycles in Infertile Obese Women: An Observational Cohort Study,” Reproductive Biomedicine Online 29 (2014): 72–79. 24813759 10.1016/j.rbmo.2014.03.006

[121] K. R. Evenson , K. C. Calhoun , A. H. Herring , D. Pritchard , F. Wen , and A. Steiner , “Association of Physical Activity in the Past Year and Immediately After In Vitro Fertilization on Pregnancy,” Fertility and Sterility 101 (2014): 1047–1054. 24524834 10.1016/j.fertnstert.2013.12.041 PMC3982290

[122] M. Rao , Z. Zeng , and L. Tang , “Maternal Physical Activity Before IVF/ICSI Cycles Improves Clinical Pregnancy Rate and Live Birth Rate: A Systematic Review and Meta‐Analysis,” Reproductive Biology and Endocrinology 16 (2018): 11. 29415732 10.1186/s12958-018-0328-z PMC5803901

[123] E. Kakargia , E. Mamalakis , M. Frountzas , E. Anagnostou , and C. Siristatidis , “The Role of Maternal Physical Activity on In Vitro Fertilization Outcomes: A Systematic Review and Meta‐Analysis,” Archives of Gynecology and Obstetrics 307 (2023): 1667–1676. 35596747 10.1007/s00404-022-06606-0

[124] A. L'Heveder , M. Chan , A. Mitra , L. Kasaven , S. Saso , and T. Prior , “Sports Obstetrics: Implications of Pregnancy in Elite Sportswomen, a Narrative Review,” Journal of Clinical Medicine 11 (2022): 4977. 36078907 10.3390/jcm11174977 PMC9456821

